# In Memoriam—Wm. M. Zerns, M. D.

**Published:** 1887-12

**Authors:** 


### IN MEMORIAM—WILLIAM M. ZERNS, M. D.

### PREAMBLE AND RESOLUTIONS OF THE BŒNNINGHAUSEN MEDICAL CLUB, OF PHILADELPHIA, ON THE DEATH OF DR. ZERNS.

William M. Zerns, M. D., of Philadelphia, having been removed from our social and medical circle by death, we hereby resolve:

1st. That we recognize in his decease the loss of a valued friend and counselor to ourselves, and to the medical profession of an honest, conscientious, and skillful physician.

2d. That we extend to his family and companions in their sad bereavement our heartfelt sympathy for the loss of a loving husband, a kind father, and a beneficent friend.

3d. That a copy of these resolutions be sent to his wife; that they be printed in the *Hahnemannian Monthly* and THE HOMŒOPATHIC PHYSICIAN, of Philadelphia, and that they be entered in the journal of this Society.

H. NOAH MARTIN, M. D.,
GEO. W. SMITH, M. D.,
*Committee.*

